# Comparison of the effects of therapeutic exercise with either an educational booklet or vitamin-D3 supplement in the management of chronic low back pain: study protocol for an assessor blinded multicenter randomized clinical trial

**DOI:** 10.12688/f1000research.127948.1

**Published:** 2022-11-21

**Authors:** Muhammad Shahidul Islam, K. M. Amran Hossain, Md. Sohrab Hossain, Rashida Parvin, Nadia Afrin Urme, Veena Raigangar, Iqbal Kabir Jahid, Md. Feroz Kabir, Md. Ashrafuzzaman Zahid

**Affiliations:** 1 Department of Nutrition & Food Technology, Jashore University of Science & Technology, Jashore, 7408, Bangladesh; 2 Department of Physiotherapy & Rehabilitation, Jashore University of Science & Technology, Jashore, 7408, Bangladesh; 3 Department of Physiotherapy, Bangladesh Health Professions Institute, Savar, 1343, Bangladesh; 4 Department of Physiotherapy, School of Sport and Health Sciences, University of Brighton, Brighton, UK; 5 Department of Microbiology, Jashore University of Science & Technology, Jashore, 7408, Bangladesh

**Keywords:** Chronic Low Back Pain, Therapeutic exercise, Vitamin-D3 supplement, Booklet

## Abstract

**Background**: It is important to know the best intervention approach to replenish serum vitamin D levels along with therapeutic interventions for chronic low back pain (CLBP) patients. From the researcher’s knowledge, no study compared “vitamin D supplement” or “booklet education on sun exposure, nutrition and lifestyle” with therapeutic exercise for CLBP cases. Researchers hypothesize that multidimensional comprehensive management of therapeutic exercise and an education booklet (TEB) on sun exposure, nutrition, and lifestyle might be superior to therapeutic exercise and oral vitamin D supplement (TED) for CLBP patients with vitamin D deficiency.

**Methods**: We planned for an assessor-blinded two-arm multicenter Randomized Clinical Trial (RCT) protocol to compare the efficacy of TEB compared to TED for CLBP patients with vitamin D deficiency at 2 months and 6 months after baseline recruitment in designated centers in Dhaka city. The primary outcome measures will include pain by Brief Pain Inventory (BPI), and serum vitamin D3 level and secondary outcome measures will include disability by Ronald Morris Disability Questionnaire (RMDQ).

**Discussion**: This study will provide evidence for an appropriate prescription for the management of CLBP patients having vitamin D deficiency.

Registration: Clinical Trials Registry India ( CTRI/2022/11/047074).

## Introduction

Chronic Low back pain (CLBP) is identified as one of the leading contributors to global disease burden. ^1^ It is a commonly prevalent musculoskeletal condition among non-communicable diseases in all countries, ranging from developing to developed countries, and in all age groups from children to the elderly population; affecting almost everyone during their lifespan. ^2^ About 55–80% of people suffer from low back pain (LBP) in their lifetime, and the worldwide yearly cost of managing chronic LBP is estimated to be a trillion dollars. ^3^ The incidence of LBP is linked to several biopsychosocial aspects, including mechanical, traumatic, pathological and degenerative causes; bone health is known to be associated with both degenerative and mechanical types of LBP. ^4^ Approximately 50% of patients seeking treatment for LBP of over 3 months’ duration are found to be additionally suffering from vitamin D and other nutritional deficiencies. ^5^ One study suggests a mean decrease of vitamin D levels may increase overall body pain. ^6^ A systematic review reported that vitamin D has the potential to decrease pain and inflammation by modifying sensory neuron excitability and anti-inflammatory and pro-inflammatory cytokines. Alongside pain remission, vitamin D levels are linked to increases in muscle strength, that contribute to improving function in patients with LBP. ^7^ A strong relationship between LBP and decreased vitamin D level are noted in elderly women, but it is still debated whether low vitamin D can predict severe LBP in the general population. ^8^ The urban population monograph is moving towards a more sedentary lifestyle and extended sitting hours with almost 12 hours spent in sedentary office jobs in Bangladesh, and this is combined with less exposure to sunshine for city dwellers. This has led to increased number of LBP cases with insufficient serum vitamin D levels in Bangladesh; those with a sedentary lifestyle and obesity form the majority of suffers. ^9^

A quasi-experimental study shows that therapeutic exercise and vitamin D supplement can be a promising treatment to battle these LBP cases, ^10^ however the study didn’t elaborate a specific protocol. Other studies suggest that aerobic exercise (low, moderate, high), stretching, balance, motor control exercises, core stability, coordination, muscular strength exercises, and flexibility programs are types of exercises that have significant outcome on LBP. But because of the intricacy of LBP, it is uncertain which of these types of exercises has the best outcome for rehabilitation; this calls for more in depth studies. ^10^^,^^11^ Research also recommend the necessity of active rehabilitation, including therapeutic exercise (TE), which is emphasized in evidence-based guidelines for the therapy of chronic low back pain (CLBP), but there is no universal agreement on the most efficient type of exercises. ^12^

Vitamin D supplementation can be provided by different approaches including natural approaches, lifestyle education and oral vitamin D supplementation. However, it has been demonstrated that engaging in any type of regular physical exercise increases circulating vitamin D and upregulates the vitamin receptor expression in muscles. ^13^ An educational booklet is an effective intervention approach for health-care professionals to deliver regular education concerning the causes, mechanisms, natural history, and prognosis of LBP, and promote the benefits of physical activity and exercise. ^14^ In previous studies, booklets on lifestyle, exercise and sun exposure ^14^^,^^15^ or exercise and vitamin D3 supplementation ^16^ have found to be effective for CLBP. Therapeutic exercise and vitamin D supplements have been found to be effective for the Dhaka city dwellers in Bangladesh, ^10^ and creating an educational booklet can be a great solution to raising awareness of CLBP with vitamin D deficiency. ^9^ Educational booklets on exercise, sun exposure and healthy nutrition have proven to be promising in other studies. ^14^^,^^15^ From the researcher’s knowledge, no study comparing the use of “vitamin D supplements” or “booklet education on sun exposure, nutrition and lifestyle” along with therapeutic exercise for CLBP cases has been done.

For this study researchers proposed a two-tailed hypothesis: use of multidimensional comprehensive management by therapeutic exercise and education booklet on sun exposure, nutrition and lifestyle (TEB) is superior to therapeutic exercise and oral vitamin D supplement (TED), for CLBP patients with vitamin D deficiency considering the following:

1. CLBP patients with vitamin D deficiency in the TEB arm show significant improvement in painful symptoms compared to the TED arm at 2 months and 6 months after baseline recruitment.
2. CLBP patients with vitamin D deficiency in the TEB arm show significant improvement in serum vitamin D levels compared to the TED arm at 2 months and 6 months after baseline recruitment.
3. CLBP patients with vitamin D deficiency in the TEB arm show significant improvement in disability status compared to the TED arm at 2 months and 6 months after baseline recruitment.

The specific objectives are:

1. To design a protocol of therapeutic exercise, along with an educational booklet on sun exposure, nutrition and lifestyle, and vitamin D supplementation for the CLBP patients with vitamin D deficiency.
2. To evaluate the effectiveness of therapeutic exercise along with education booklet on sun exposure, nutrition and lifestyle, on painful symptoms, serum vitamin D level and disability for CLBP patients with vitamin D deficiency at 2 months and 6 months’ post-test compared to baseline.
3. To explore the effectiveness of therapeutic exercise along with oral vitamin D supplement on painful symptoms, serum vitamin D level and disability for CLBP patients with vitamin D deficiency at 2 months and 6 months post-test compared to baseline.
4. To study the comparative effectiveness of both groups on painful symptoms, serum vitamin D levels and disability for CLBP patients with vitamin D deficiency at 2 months and 6 months’ post-test compared to baseline.

## Methods

Researchers plan for an assessor blinded two arm multicenter Randomized Clinical Trial (RCT) protocol to compare the efficacy of therapeutic exercise and education booklet on sun exposure, nutrition and lifestyle versus therapeutic exercise and oral vitamin D supplement for CLBP patients with vitamin D deficiency at 2 months and 6 months after baseline recruitment in designated rehabilitation centers in Dhaka city.

For this potential trial, researchers will follow Standard Protocol Items: Interventional Trials 2013 (SPIRIT) guidelines, to help ensure quality of the interventional trial ( Table 1).

**Table 1.** Study protocols according to SPIRIT guidelines.

Study (Status)	Teams (Preparation)			Patients (Execution)			
	Preparation & planning	Training to team	Piloting	Enroll	Study		
Time					Baseline 0	2 months	6 months
**Intervention**	×	×	×		×	×	
**Enrollment**	×		×	×			
Informed Consent	×		×	×			
Eligibility	×	×		×			
**Evaluations**							
BPI		×	×		×	×	×
Vit. D3		×	×		×	×	×
RMDQ		×	×		×	×	×

Abbreviations: BPI, Brief Pain Inventory; Vit. D3, Serum 25(OH)D; RMDQ, Roland Morris Disability Questionnaire.

### Study setting

To meet the objectives of the trial and prevent trial contamination, the experimental group interventions will take place at the Centre for the Rehabilitation of the Paralysed (CRP) and control group interventions will take place at SAIC College of Medical Science & Technology. We expect to get cases with similar geographical and baseline criteria of city dwellers having CLBP. Data collection from different sites will increase the generalizability of the study and prevent cross-contamination of data.

### Eligibility criteria

Eligibility criteria for selecting participants include having (1) CLBP for more than 3 months and central in nature as defined by ICD-10-CM-Code M54.5 criteria, (2) vitamin D deficiency determined by a serum 25(OH) D3 level of less than 20 ng/mL, ^16^ (3) living or working in Dhaka city in any office, industry and corporate setting with a static posture or desk job with at least 6 hours of sitting per day for an average of 22 days per month, (4) age 18 years and above, both genders. Participants that provide informed consent for the study and interventions will be recruited in the study. On the other hand, participants will be excluded if (1) there is presence of any kind of comorbidity that can affect serum vitamin D level like RA, Ankylosing Spondylitis, Osteomalacia, TB spine, etc., and any history of osteoporotic fracture, (2) women more than 50 years or reported post-menopausal women, ^14^ (3) use of calcium, vitamin D3 supplements, resistance training, and high impact weight-bearing activities regularly within the past 6 months, (4) low back pain patients with Dural sign, positive straight leg raise test, or bowel and bladder incontinence and (5) withdrawal of participation during 8 weeks intervention. Participants who are already involved in another study will not be considered for study.

### Interventions

Participants will receive intervention ( Table 2) as per the registered protocol for therapeutic exercise along with advice by booklet, or therapeutic exercise and Vitamin D3 supplement. ^17^

**Table 2.** Intervention details.

Code		Interventions
**B**		**Contents:** ✓ Avoid long time static sitting and standing. It is recommended that standing work be interspersed with periods of sitting work and vice versa. ^18^ ✓ Take naturally vitamin D3 containing foods like milk, cereal, orange juice, yogurt, cereal, mushroom, margarine, hard-boiled eggs, sea fish like tuna, salmon, etc. ^19^ ✓ 30-35 minutes of sun exposure between 11 am to 2 pm. ^15^^,^^20^ ✓ 7–8-hours of sleep per night. ^21^ ✓ Avoid stress, smoking and maintain healthy body weight. ^22^ ✓ Avoid alcohol consumption. ✓ Do regular physical activity.
**TE**	**TH.1**	**Therapeutic exercise:** Postural advice: maintain erect posture in sitting and standing, avoid long periods of sitting or standing, avoid forward bending. ^23^
	**TH.2**	McKenzie's directional preference includes sustained positioning, flexion, or extension principle (most often need extension principle). Exercises will be completed in a set of 10 repetitions. ^24^
	**TH.3**	Stretching exercises: stretching of erector spine muscle, hamstring, and triceps surae. ^25^
	**TH.4**	Lumbar stabilization exercises. ^26^
	**TH.5**	Weight-bearing aerobics exercise includes jogging, jogging with stair climbing, and 30 minutes of brisk walking. ^7^
	**TH.6**	Heating modalities: infra-red radiation.
**D**		40,000 IU vitamin D3 capsule prescribed by registered physician made by a specific pharmaceutical company of Bangladesh per week for 8 weeks. ^10^

Abbreviations: B, Booklet; TH, Therapeutic exercise; S, Vitamin D Oral Supplement.

**Booklet**

Using the created educational booklet, the physician will provide guidelines for CLBP patients with low vitamin D levels to raise awareness regarding appropriate diet and healthy lifestyle, back care and advise to increase nutritional status as well as overall physical function to minimize disability.

**Therapeutic exercise**

Both groups will receive therapeutic exercises. Exercises will focus on both back pain and disability minimization of the participants. Each session will last for 25-30 minutes, 4 days per weeks and for 8 weeks. The progression of therapeutic exercise will be as per the registered protocol. ^17^

**Vitamin D3 supplement**

One group will receive a vitamin-D3 supplement along with therapeutic exercise, which will be provided by a research fund. The patients will be given 40,000 IU vitamin D3 capsule made by a specific pharmaceutical company of Bangladesh per week for 8 weeks. ^10^ A physician will fix the dose, provide a chart of capsule-taking dates and time, a record of this will be kept to avoid any kind of misconduct.

We expect there will be no major adverse effect for therapeutic exercise and booklet group and that there will be no request of dosage change or worsening of patients’ condition. If any of these occur we will discuss with the patient. If the patient is not willing not to continue, we will stop the intervention, and keep the data for intention to treat analysis. The vitamin D supplement group may experience some adverse effect; we will manage as per the standard measures described in the “safety measures” section. The adherence to these interventions will be monitored through checklist (Extended data 1 ^31^). We will also monitor adverse effect using a checklist (Extended data 2 ^31^).

**Outcome measurement**

***Primary outcome***

*Pain*

In this study, the Brief Pain Inventory (BPI) will be used to measure pain. The BPI is a patient-reported measurement scale that assesses the pain severity, influence of pain on daily function, site of pain, information about pain medication, and quantity of pain relief in the preceding 24 hours. ^27^ This is an 11-point numerical rating scale rated from 0 to 10, where 0 denotes “no pain” and 10 denotes “pain as bad as you can imagine”. A patient is asked to score his/her perceived pain intensity on a scale for the items as worse, least, average in the preceding 24 hours, and worse at right now. BPI has excellent internal consistency for pain severity (Cronbach’s alpha 0.78 to 0.96) and pain interference (Cronbach’s alpha 0.83 to 0.95), and also has good validity and reliability. ^10^

*Vitamin-D3 level*

Serum 25(OH) D will be used to measure the level of Vitamin D3. Tests will be advised by an expert physician, and researchers will collect information from laboratory test reports. Patients will be categorized based on vitamin D levels, such as deficient (less than 20 ng/mL); insufficient (21 to 29 ng/mL); and sufficient (more than 30 to 100 ng/mL). ^10^^,^^20^

***Secondary outcome***

*Disability*

Roland Morris Disability Questionnaire (RMDQ) will be used to measure disability. RMDQ was developed in order to explain the natural history of back pain. It is a 24-item questionnaire where patients' rated scores might range from “0” (no disability) to “24” (severe disability). For each of the 24 questions that were marked by patients, it counted as a score of 1-point. ^28^ This study will use the Bengali version of RMDQ. It has excellent internal consistency (Cronbach’s alpha = 0.89), test-retest reliability (ICC = 0.95), and validity. ^29^

## Participant timeline

### Sample size

As the sample of the study will be collected by a hospital-based randomization technique, sample size estimations won’t be done. One study supports the idea that a standard calculation for low back pain trials with not less than 152 participants can obtain significant superiority changes with an alpha value.05, 80% power and 95% confidence interval. ^30^

### Randomization

Researchers plan for hospital-based randomization in both study centers from 1 ^st^ December 2022 to 30 ^th^ May 2023 by sequential random sampling and eligibility screening. For concealed allocation, a computer based random sequence maintained by independent and blinded person out of the research team will be prepared and protocols will be concealed into envelopes. The treatment providers and the patients will also be blinded as to whether they are in control or experimental group.

### Recruitment and study procedure

**Figure 1.**
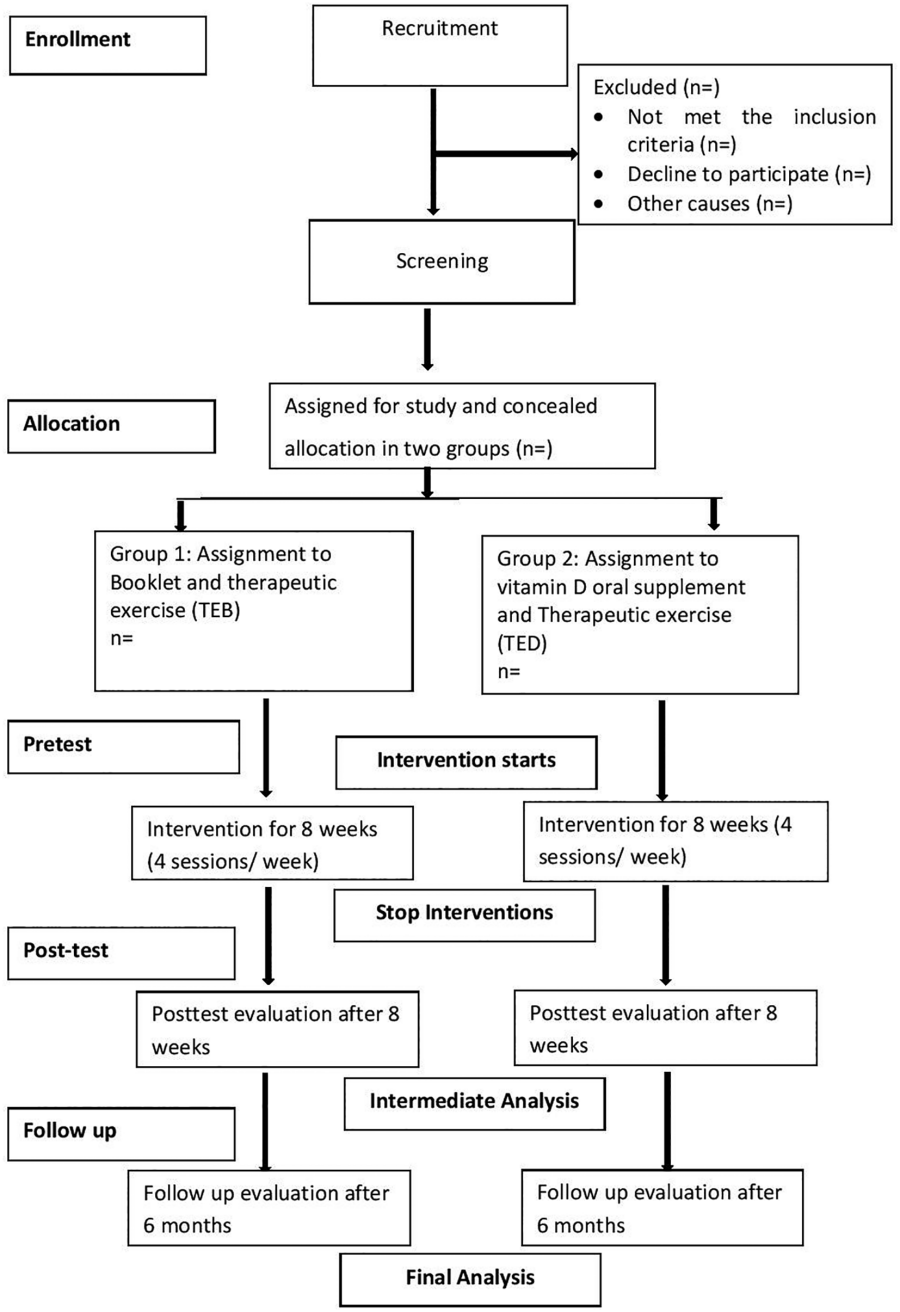
CONSORT diagram.

We will follow the Consolidated Standards of Reporting Trials (CONSORT) to maintain the standards of the study procedure ( Figure 1). For the initial recruitment the LBP patients attending outdoor clinics of both centers from 1st December 2022 to 30th May 2022 will be primarily screened by the outdoor team and provided participant information sheet (PIS) of the study. The final screening will be performed by the trained primary assessors, one per study setting to check the inclusion and exclusion criteria and provide the subject an ID number, if eligible. The patient will meet the blinded assessor who will then take pretest data in a separate room, and collect a blood sample, before returning the patient to the outdoor pool. From outdoor pool, the patient will have a concealed envelop with another random ID number matched by the initial ID number given and meet the intervention provider (physiotherapist and physician or physiotherapist alone). Patient will receive the intervention provided in the written guideline enclosed in the concealed envelope. After 8 weeks of treatment completion, the patient will be further screened by blinded assessor, and another blood sample will be collected before discharge. After six months, the patient will be invited to the treatment center or visited in their house or workplace for follow up evaluation and blood sample collection. Therapeutic exercise will be provided by a graduate physiotherapist, and medication will be provided by a registered medical practitioner. Patient will pay for the physiotherapy treatment sessions but will not pay for any additional blood tests, medication or booklet.

### Monitoring

Patients will be monitored during the intervention session, and the medication chart and home exercise checklist (Extended data 1 ^31^) will be maintained for recording the interventions. Patient data will be reviewed by a team from a different organization out of the study setting. The completed forms and questionnaire, along with blood report, will be evaluated by the monitoring team. Any kind of change or modifications to the methodology and intervention protocol will be communicated to the Ethics Committees. The research team will have access to the data and interim results and be in charge of making the final decision to change or end the study, hence carrying out interim analysis.

### Safety measures to avoid harmful effects

Although it is expected that vitamin D3 supplementation and therapeutic exercise will not produce harmful effects on patients, patients should be instructed to inform the physician and physiotherapist if they feel any kind of discomfort (including-gastrointestinal, skin, musculoskeletal problem etc.) after the intervention. Before starting, the physiotherapist and physician will screen patients for any contraindications to intervention. If any serious harmful effects are found, researchers will report this during the final publication. The adverse effects reporting checklist will be provided during intervention (Extended data 2 ^31^).

### Data analysis

Data will be analyzed based on its nature. Calculation and data auditing will be done using Microsoft Excel 2016. Data will be analyzed by SPSS version 23, and R-4.2.1 for Windows. Eligibility for parametric analysis will be checked using bell’s curve, skewness, kurtosis, Kolmogorov–Smirnov test and Shapiro–Wilk test. Continuous variables will be represented by using an arithmetic mean and standard deviation. Categorical data will be represented by percentage (%) and frequency. Baseline compatibility will be checked by Pearson correction or chi-square test. For parametric data, the pretest to posttest between group analyses will be conducted through an independent t test and within group analyses through paired sample t tests. For non-parametric data, non-parametric alternative tests like the Wilcoxon Signed Rank test and Mann Whitney U-test will be performed. For the analysis of treatment superiority among three measurements, one-way ANOVA or Friedman’s ANOVA will be used with post-hoc analysis. The level of significance is set as an alpha value <05. We may include intention to treat analysis based on the situation of data collection.

### Ethical issues and informed consent

According to ethical guidelines, the researchers will abide by the Helsinki declaration. The participants' participation will be entirely voluntary, and they will have the right to withdraw from the trial at any time during the trial. The participants will be assured that participation in or withdrawal from the study will not cause any change to their regular treatment program. Participants will sign the informed consent (Extended data 3 ^31^). The Institute of Physiotherapy Rehabilitation and Research of the Bangladesh Physiotherapy Association (BPA) has provided ethical permission (BPA-IPRR/IRB/06/16/2060) on 16 ^th^ June 2022 to proceed with the study (Extended data 4 ^31^). The trial has been registered with Clinical Trials Registry India ( CTRI/2022/11/047074) (Extended data 5 ^31^). In case of any changes to the protocol, research team will notify to Institutional review board, the trial registry platform and in the later publications. The personal information of the participants will be confidential and stored unanimously in a dataset at the Department of nutrition and food technology at Jashore university of Science & Technology.

### Study status

This study has concluded the assignment of health clinics, training of intervention provider, ethical approval and applied for trial registration. We anticipate beginning this trial on 1 ^st^ December 2022.

## Discussion

There is an increasing concern of LBP and vitamin D deficiency for chronic pain suffers that is leading the working people towards disability and inefficiency to work. ^5^^,^^9^ A non-randomized quasi experimental study ^10^ found therapeutic exercise and vitamin D oral supplementation is effective to reduce pain, replenish vitamin D3 level with short term results. Our study will meet the necessity of randomized systematic evaluation of therapeutic exercises and vitamin D supplement compensation in two different approaches, either by sun exposure, nutrition and healthy lifestyle or by taking oral supplements. We will evaluate outcome in both short term (2 months) and long-term effect after 6 months of stopping the intervention. The experimental group is the therapeutic exercise and booklet group because we assume a positive lifestyle and exercise can replace the role of oral medication supplement, as these were derived as a predictor in observational studies. ^9^

The methodological standard of the proposed trial adheres to the Enhancing the QUAlity and Transparency Of health Research (EQUATOR) guidelines to ensure the rigor of the study. As this is a two tailed hypothesis, we assume any treatment can be superior or both may have similar effect. The similar effect is also a positive finding, because oral vitamin D supplement have some adverse effects if taken for longer durations. ^12^ Moreover, if the study would have four arms including two interventions, a group with only vitamin D supplement and another with therapeutic exercise and a placebo vitamin D supplement, that could ensure true effects. However, researchers had to limit the study considering funding, scope of practice and complicated management issues. As outcome indicators pain and vitamin D3 levels will be used as primary outcomes and disability as secondary outcome, because previous research suggests disability as a consequence. ^18^ BPI measures not only pain severity, but also pain affective interference and pain physical interference, ^10^^,^^27^ that is consistent to the effect of intervention.

The future direction of the study can explain the importance of therapeutic exercise and their role upon the production, absorption, deposition and function of serum vitamin D level for CLBP cases. Evidence shows that exercise improves the metabolic functions of vitamin D and reduces musculoskeletal pain by modifying sensory neuron excitability and anti-inflammatory and pro-inflammatory cytokines, ^7^ thus contributing to the remission of disability. In this study, if education impacts lifestyle changes and has a positive impact, it will lead to further investigation on how the body can self-regulate vitamin D3 level and add to self-remission of CLBP. Also, this study may have a positive outcome on the recurrence of LBP or delaying the episodic pattern of pain in CLBP patients.

## Author contributions

MSI, KMAH, MAZ contributed to Conceptualizing, Planning, Funding Acquisition, Investigation, Administration, Writing (review & editing), and approval. MSH, RP, IKJ, MFK contributed to Investigation, Conceptualizing, Supervision, and review. VR, NAU contributed to Conceptualizing, Writing (review &editing), and approval.

## Data accessibility

### Underlying data

No underlying data are associated with this article.

### Extended data

Mendeley Data: Therapeutic Exercise & Vitamin D for CLBP. https://doi.org/10.17632/d4hf2hjjxr.2. ^31^

This project contains the following extended data:

- Extended data 1: the medication chart and home exercise checklist
- Extended data 2: the adverse effects reporting checklist
- Extended data 3: Informed consent
- Extended data 4: Institutional Review Board (IRB) permission
- Extended data 5: Clinical Trial Registry
- Extended data 6: SPIRIT Checklist

Data are available under the terms of the Creative Commons Attribution 4.0 International license (CC-BY 4.0).

## Reporting guidelines

For generating the protocol, we followed the Interventional Trials 2013 (SPIRIT) guidelines (Table 1), and for the RCT we will follow Consolidated statements for reporting randomized trials (CONSORT) guideline (Figure 1).

Mendeley Data: SPIRIT checklist for ‘Comparison of the effects of therapeutic exercise with either an educational booklet or vitamin-D3 supplement in the management of chronic low back pain: study protocol for an assessor blinded multicenter randomized clinical trial’. https://doi.org/10.17632/d4hf2hjjxr.2. ^31^

Data are available under the terms of the Creative Commons Attribution 4.0 International license (CC-BY 4.0).

## References

[1] Froud R Patterson S Eldridge S : A systematic review and meta-synthesis of the impact of low back pain on people’s lives. *BMC Musculoskelet. Disord.* 2014;15(1):1–4. 24559519 10.1186/1471-2474-15-50 PMC3932512

[2] Hoy D Bain C Williams G : A systematic review of the global prevalence of low back pain. *Arthritis Rheum.* 2012;64(6):2028–2037. 10.1002/art.34347 22231424

[3] Pan CC Simon P Espinoza Orías AA : Lumbar facet joint subchondral bone density in low back pain and asymptomatic subjects. *Skelet. Radiol.* 2020;49(4):571–576. 10.1007/s00256-019-03314-w 31673719 PMC7024659

[4] Briggs AM Straker LM Burnett AF : Chronic low back pain is associated with reduced vertebral bone mineral measures in community-dwelling adults. *BMC Musculoskelet. Disord.* 2012;13(1):1–10. 22458361 10.1186/1471-2474-13-49 PMC3359205

[5] Correia MI Hegazi RA Higashiguchi T : Evidence-based recommendations for addressing malnutrition in health care: an updated strategy from the feedM. E. Global Study Group. *J. Am. Med. Dir. Assoc.* 2014;15(8):544–550. 10.1016/j.jamda.2014.05.011 24997720

[6] Wu Z Malihi Z Stewart AW : The association between vitamin D concentration and pain: a systematic review and meta-analysis. *Public Health Nutr.* 2018;21(11):2022–2037. 10.1017/S1368980018000551 29559013 PMC10260782

[7] Zadro JR Shirley D Ferreira M : Is Vitamin D Supplementation Effective for Low Back Pain? A Systematic Review and Meta-Analysis. *Pain Physician.* 2018;21(2):121–145. 29565945

[8] Hao-Wei X Shu-Bao Z Yu-Yang Y : Relationship between vitamin D and nonspecific low back pain may be mediated by inflammatory markers. *Pain Physician.* 2021;24(7):1015. 34704712

[9] Ali M Uddin Z : Factors associated with vitamin D deficiency among patients with musculoskeletal disorders seeking physiotherapy intervention: a hospital-based observational study. *BMC Musculoskelet. Disord.* 2022;23(1):1–9. 36042435 10.1186/s12891-022-05774-z PMC9426039

[10] Ali M Uddin Z Hossain A : Combined Effect of Vitamin D Supplementation and Physiotherapy on Reducing Pain Among Adult Patients With Musculoskeletal Disorders: A Quasi-Experimental Clinical Trial. *Front. Nutr.* 2021;8:717473. 10.3389/fnut.2021.717473 34676231 PMC8523800

[11] Gordon R Bloxham S : A systematic review of the effects of exercise and physical activity on non-specific chronic low back pain. *In Healthcare.* 2016;4(2):22. 10.3390/healthcare4020022 27417610 PMC4934575

[12] Pardo GB Girbés EL Roussel NA : Pain neurophysiology education and therapeutic exercise for patients with chronic low back pain: a single-blind randomized controlled trial. *Arch. Phys. Med. Rehabil.* 2018;99(2):338–347. 10.1016/j.apmr.2017.10.016 29138049

[13] Holick MF : Sunlight and vitamin D for bone health and prevention of autoimmune diseases, cancers, and cardiovascular disease. *Am. J. Clin. Nutr.* 2004;80(6):1678–1688. 10.1093/ajcn/80.6.1678S 15585788

[14] Simula AS Jenkins HJ Holopainen R : Transcultural adaption and preliminary evaluation of “understanding low back pain” patient education booklet. *BMC Health Serv. Res.* 2019;19(1):1–1. 31888605 10.1186/s12913-019-4854-y PMC6936060

[15] Augustine LF Madhavan KN Kulkarni B : Optimal duration of sun exposure for adequate cutaneous synthesis of vitamin D in Indian cities: an estimate using satellite-based ultraviolet index data. *Biomed. J. Sci. Tech. Res.* 2018;6:5073–5077.

[16] Lakkireddy M Karra ML Patnala C : Efficiency of vitamin D supplementation in patients with mechanical low back ache. *J. Clin. Orthop. Trauma.* 2019;10(6):1101–1110. 10.1016/j.jcot.2019.06.018 31708636 PMC6834986

[17] Islam MS Zahid MA Hossain MS : Effect of Therapeutic Exercise, Educational booklet and Vitamin D3 Supplement for the Management of Chronic Mechanical Low Back Pain Muhammad Shahidul Islam, Dr. Md. Ashrafuzzaman Zahid, Professor Dr. Md. Sohrab Hossain, protocols.io. 2022. 10.17504/protocols.io.rm7vzyqz5lx1/v1 PMC13044536 41937888

[18] Gallagher KM Campbell T Callaghan JP : The influence of a seated break on prolonged standing induced low back pain development. *Ergonomics.* 2014;57(4):555–562. 10.1080/00140139.2014.893027 24734970

[19] Zaman S Hawlader MD Biswas A : High prevalence of vitamin D deficiency among Bangladeshi children: an emerging public health problem. *Health.* 2017;09(12):1680–1688. 10.4236/health.2017.912123

[20] Harinarayan CV Holick MF Prasad UV : Vitamin D status and sun exposure in India. *Dermato-endocrinology.* 2013;5(1):130–141. 10.4161/derm.23873 24494046 PMC3897581

[21] Gao Q Kou T Zhuang B : The association between vitamin D deficiency and sleep disorders: a systematic review and meta-analysis. *Nutrients.* 2018;10(10):1395. 10.3390/nu10101395 30275418 PMC6213953

[22] Dean E Söderlund A : What is the role of lifestyle behaviour change associated with non-communicable disease risk in managing musculoskeletal health conditions with special reference to chronic pain? *BMC Musculoskelet. Disord.* 2015;16(1):1–7. 25888381 10.1186/s12891-015-0545-y PMC4397667

[23] Kim D Cho M Park Y : Effect of an exercise program for posture correction on musculoskeletal pain. *J. Phys. Ther. Sci.* 2015;27(6):1791–1794. 10.1589/jpts.27.1791 26180322 PMC4499985

[24] Dunsford A Kumar S Clarke S : Integrating evidence into practice: use of McKenzie-based treatment for mechanical low back pain. *J. Multidiscip. Healthc.* 2011;4:393. 22135496 10.2147/JMDH.S24733 PMC3215349

[25] França FR Burke TN Caffaro RR : Effects of muscular stretching and segmental stabilization on functional disability and pain in patients with chronic low back pain: a randomized, controlled trial. *J. Manip. Physiol. Ther.* 2012;35(4):279–285. 10.1016/j.jmpt.2012.04.012 22632587

[26] Yoon JS Lee JH Kim JS : The effect of swiss ball stabilization exercise on pain and bone mineral density of patients with chronic low back pain. *J. Phys. Ther. Sci.* 2013;25(8):953–956. 10.1589/jpts.25.953 24259892 PMC3820231

[27] Farrar JT Young JP Jr LaMoreaux L : Clinical importance of changes in chronic pain intensity measured on an 11-point numerical pain rating scale. *Pain.* 2001;94(2):149–158. 10.1016/S0304-3959(01)00349-9 11690728

[28] Jordan K Dunn KM Lewis M : A minimal clinically important difference was derived for the Roland-Morris Disability Questionnaire for low back pain. *J. Clin. Epidemiol.* 2006;59(1):45–52. 10.1016/j.jclinepi.2005.03.018 16360560

[29] Islam SM Emran M Baral AB : Roland Morris disability questionnaire in Bengali for evaluation of patients with low back pain. *KYAMC J.* 2020;11(1):21–25. 10.3329/kyamcj.v11i1.47146

[30] Froud R Rajendran D Patel S : The power of low back pain trials: a systematic review of power, sample size, and reporting of sample size calculations over time, in trials published between 1980 and 2012. *Spine.* 2017;42(11):E680–E686. 10.1097/BRS.0000000000001953 27792111

[31] Hossain KMA Zahid MA Islam MS : Therapeutic Exercise & Vitamin D for CLBP. *Mendeley Data.* 2022;V2. 10.17632/d4hf2hjjxr.2

