# Comparison of the effects of therapeutic exercise with either an educational booklet or vitamin-D3 supplement in the management of chronic low back pain: study protocol for an assessor blinded multicenter randomized clinical trial

**DOI:** 10.12688/f1000research.127948.2

**Published:** 2026-03-03

**Authors:** Muhammad Shahidul Islam, K. M. Amran Hossain, Md. Sohrab Hossain, Rashida Parvin, Nadia Afrin Urme, Veena Raigangar, Iqbal Kabir Jahid, Md. Feroz Kabir, Md. Ashrafuzzaman Zahid

**Affiliations:** 1 Department of Nutrition & Food Technology, Jashore University of Science & Technology, Jashore, 7408, Bangladesh; 2 Department of Physiotherapy & Rehabilitation, Jashore University of Science & Technology, Jashore, 7408, Bangladesh; 3 Department of Physiotherapy, Bangladesh Health Professions Institute, Savar, 1343, Bangladesh; 4 Department of Physiotherapy, School of Sport and Health Sciences, University of Brighton, Brighton, UK; 5 Department of Microbiology, Jashore University of Science & Technology, Jashore, 7408, Bangladesh

**Keywords:** Chronic Low Back Pain, Therapeutic exercise, Vitamin-D3 supplement, Booklet

## Abstract

**Background**: Chronic low back pain (CLBP) is a common musculoskeletal disorder that significantly impairs quality of life and is frequently associated with vitamin D deficiency. Therapeutic exercise is a cornerstone of management, yet the optimal adjunct intervention to restore serum vitamin D levels remains uncertain. No prior randomized trial has compared oral vitamin D supplementation with lifestyle education on sun exposure, nutrition, and physical activity, both combined with therapeutic exercise. We hypothesize that therapeutic exercise plus an education booklet (TEB) may be superior to therapeutic exercise plus oral vitamin D supplementation (TED) in reducing pain and disability while improving serum vitamin D levels.

**Methods**: This assessor-blinded, two-arm, multicenter randomized controlled trial (RCT) will be conducted in hospitals and rehabilitation centers in Dhaka city, Bangladesh. Adult participants (18–65 years) with CLBP and confirmed vitamin D deficiency will be recruited and randomized to either TEB or TED groups. Both groups will receive standardized therapeutic exercise protocols. The intervention duration will be 6 months, with assessments at baseline, 2 months, and 6 months. Primary outcomes include pain intensity measured by the Brief Pain Inventory (BPI) and serum 25(OH)D3 levels. Secondary outcomes include disability assessed by the Roland-Morris Disability Questionnaire (RMDQ). Statistical analysis will follow the intention-to-treat principle, using repeated measures ANOVA to compare group differences over time.

**Discussion**: This trial will provide evidence on whether a multidimensional lifestyle-based education approach combined with therapeutic exercise offers greater benefit than vitamin D supplementation with exercise in CLBP patients with vitamin D deficiency. The findings will inform clinical guidelines and support comprehensive management strategies for CLBP, particularly in resource-limited settings.

Registration: Clinical Trials Registry India ( CTRI/2022/11/047074).

## Introduction

Chronic Low back pain (CLBP) is identified as one of the leading contributors to global disease burden. ^1^ It is a commonly prevalent musculoskeletal condition among non-communicable diseases in all countries, ranging from developing to developed countries, and in all age groups from children to the elderly population; affecting almost everyone during their lifespan. ^2^ About 55–80% of people suffer from low back pain (LBP) in their lifetime, and the worldwide yearly cost of managing chronic LBP is estimated to be a trillion dollars. ^3^ The incidence of LBP is linked to several biopsychosocial aspects, including mechanical, traumatic, pathological and degenerative causes; bone health is known to be associated with both degenerative and mechanical types of LBP. ^4^ Approximately 50% of patients seeking treatment for LBP of over 3 months’ duration are found to be additionally suffering from vitamin D and other nutritional deficiencies. ^5^ One study suggests a mean decrease of vitamin D levels may increase overall body pain. ^6^ A systematic review reported that vitamin D has the potential to decrease pain and inflammation by modifying sensory neuron excitability and anti-inflammatory and pro-inflammatory cytokines. Alongside pain remission, vitamin D levels are linked to increases in muscle strength, which contribute to improving function in patients with LBP. ^7^ A strong relationship between LBP and decreased vitamin D levels is noted in elderly women. However, it is still debated whether low vitamin D can predict severe LBP in the general population. ^8^ The urban population monograph is moving towards a more sedentary lifestyle and extended sitting hours with almost 12 hours spent in sedentary office jobs in Bangladesh, and this is combined with less exposure to sunshine for city dwellers. This has led to an increased number of LBP cases with insufficient serum vitamin D levels in Bangladesh; those with a sedentary lifestyle and obesity form the majority of sufferers. ^9^

A quasi-experimental study shows that therapeutic exercise and vitamin D supplements can be a promising treatment to battle these LBP cases ^10^; however the study didn’t elaborate a specific protocol. Other studies suggest that aerobic exercise (low, moderate, high), stretching, balance, motor control exercises, core stability, coordination, muscular strength exercises, and flexibility programs are types of exercises that have a significant outcome on LBP. But because of the intricacy of LBP, it is uncertain which of these types of exercises has the best outcome for rehabilitation; this calls for more in depth studies. ^10^^,^^11^ Research also recommend the necessity of active rehabilitation, including therapeutic exercise (TE), which is emphasized in evidence-based guidelines for the therapy of CLBP, but there is no universal agreement on the most efficient type of exercises. ^12^

Vitamin D supplementation can be provided by different approaches including natural approaches, lifestyle education and oral vitamin D supplementation. However, it has been demonstrated that engaging in any type of regular physical exercise increases circulating vitamin D and upregulates the vitamin receptor expression in muscles. ^13^ An educational booklet is an effective intervention approach for health-care professionals to deliver regular education concerning the causes, mechanisms, natural history, and prognosis of LBP, and promote the benefits of physical activity and exercise. ^14^ In previous studies, booklets on lifestyle, exercise and sun exposure ^14^^,^^15^ or exercise and vitamin D3 supplementation ^16^ have found to be effective for CLBP. Therapeutic exercise and vitamin D supplements are effective for the Dhaka city dwellers in Bangladesh, ^10^ and creating an educational booklet can be a great solution to raising awareness of CLBP with vitamin D deficiency. ^9^ Educational booklets on exercise, sun exposure and healthy nutrition have proven to be promising in other studies. ^14^^,^^15^ From the researcher’s knowledge, no study comparing the use of “vitamin D supplements” or “booklet education on sun exposure, nutrition and lifestyle” along with therapeutic exercise for CLBP cases has been done.

Chronic low back pain (CLBP) has been associated with vitamin D deficiency, which affects calcium metabolism, bone health, and muscle function. Deficiency may lead to osteomalacia, muscle weakness, and impaired neuromuscular control, increasing spinal instability and pain. Additionally, low vitamin D levels can elevate pro-inflammatory cytokines, intensifying pain sensitivity and contributing to chronic musculoskeletal disorders. ^8^ Clinical studies have reported a higher prevalence of vitamin D deficiency among patients with CLBP compared to healthy controls, suggesting a potential association between deficiency and pain severity. ^17, 18^ Vitamin D supplementation has been explored as a supportive measure in CLBP due to its role in bone health, muscle strength, and inflammation regulation. ^19^ Correcting deficiency may improve musculoskeletal function and reduce pain sensitivity, with clinical trials reporting symptomatic improvement in deficient patients. ^20, 21^ However, meta-analyses indicate limited overall efficacy, suggesting benefits may be restricted to those with confirmed deficiency, while excessive intake carries risks such as hypercalcemia and kidney stones. ^22^

This study hypothesizes that multidimensional comprehensive management through therapeutic exercise combined with an education booklet on sun exposure, nutrition, and lifestyle (TEB) will be superior to therapeutic exercise with oral vitamin D supplementation (TED) in patients with chronic low back pain and vitamin D deficiency. Specifically, it is expected that participants in the TEB group will demonstrate greater improvements in pain symptoms, serum vitamin D levels, and disability status compared to those in the TED group, with these effects observed at both 2 and 6 months following baseline recruitment. Following the study hypothesis, this trial holds clear clinical significance: for clinicians, it offers evidence to guide treatment choices between exercise combined with education or vitamin D3; for patients, it evaluates accessible, low-cost strategies that may improve pain and function; and for researchers, it provides rigorous comparative data to inform future studies on scalable interventions for CLBP.

The specific objectives are:

1. To design a protocol of therapeutic exercise, along with an educational booklet on sun exposure, nutrition and lifestyle, and vitamin D supplementation for the CLBP patients with vitamin D deficiency.
2. To evaluate the effectiveness of therapeutic exercise along with an education booklet on sun exposure, nutrition and lifestyle, on painful symptoms, serum vitamin D level and disability for CLBP patients with vitamin D deficiency at 2 months and 6 months post-test compared to baseline.
3. To explore the effectiveness of therapeutic exercise along with oral vitamin D supplement on painful symptoms, serum vitamin D level and disability for CLBP patients with vitamin D deficiency at 2 months and 6 months post-test compared to baseline.
4. To study the comparative effectiveness of both groups on painful symptoms, serum vitamin D levels and disability for CLBP patients with vitamin D deficiency at 2 months and 6 months’ post-test compared to baseline.

## Methods

Researchers plan for an assessor blinded two arm multicenter Randomized Clinical Trial (RCT) protocol to compare the efficacy of therapeutic exercise and an education booklet on sun exposure, nutrition and lifestyle versus therapeutic exercise and oral vitamin D supplement for CLBP patients with vitamin D deficiency at 2 months and 6 months after baseline recruitment in designated rehabilitation centers in Dhaka city.

For this potential trial, researchers will follow Standard Protocol Items: Interventional Trials 2013 (SPIRIT) guidelines, to help ensure quality of the interventional trial ( Table 1).

**Table 1.** Study protocols according to SPIRIT guidelines.

Study (Status)	Teams (Preparation)			Patients (Execution)			
	Preparation & planning	Training to team	Piloting	Enroll	Study		
Time					Baseline 0	2 months	6 months
**Intervention**	×	×	×		×	×	
**Enrollment**	×		×	×			
Informed Consent	×		×	×			
Eligibility	×	×		×			
**Evaluations**							
BPI		×	×		×	×	×
Vit. D3		×	×		×	×	×
RMDQ		×	×		×	×	×

Abbreviations: BPI, Brief Pain Inventory; Vit. D3, Serum 25(OH)D; RMDQ, Roland Morris Disability Questionnaire.

### Study setting

To meet the objectives of the trial and prevent trial contamination, the experimental group interventions will take place at the Centre for the Rehabilitation of the Paralysed (CRP) and control group interventions will take place at SAIC College of Medical Science & Technology. We expect to get cases with similar geographical and baseline criteria of city dwellers having CLBP. Data collection from different sites will increase the generalizability of the study and prevent cross-contamination of data.

### Eligibility criteria

Participants will be included if they present with chronic low back pain (CLBP) of central origin, persisting for more than three months and classified under ICD-10-CM Code M54.5, with documented vitamin D deficiency defined as serum 25(OH)D3 levels below 20 ng/mL. ^16^ Eligible individuals must be adults aged 18 years or older, of either gender, residing or working in Dhaka city in office, industry, or corporate settings that involve static postures or desk jobs requiring at least six hours of sitting per day for an average of 22 days per month, and must provide informed consent. Exclusion criteria comprise comorbid conditions that may influence vitamin D metabolism or bone health, including rheumatoid arthritis, ankylosing spondylitis, osteomalacia, tuberculosis of the spine, or a history of osteoporotic fracture. Women over 50 years of age or those who are post-menopausal will also be excluded, ^14^ as will individuals with prior use of calcium or vitamin D3 supplements, resistance training, or high-impact weight-bearing activities within the past six months. Patients presenting with neurological red flags such as dural signs, positive straight leg raise test, or bowel/bladder incontinence will not be considered. Additional exclusions include current participation in another clinical study and withdrawal during the 8-week intervention period.

### Interventions

Participants will receive interventions according to the registered study protocol, consisting of either therapeutic exercise combined with an educational booklet (TEB) or therapeutic exercise combined with oral vitamin D3 supplementation (TED). ^23^

**Booklet contents (B):** ^24– 26^

The educational booklet provides lifestyle and self-management advice designed to complement therapeutic exercise. Key recommendations include:

• Avoid prolonged static sitting or standing; alternate between sitting and standing during work.
• Consume natural sources of vitamin D3 such as milk, yogurt, fortified cereals, orange juice, mushrooms, margarine, hard-boiled eggs, and sea fish (e.g., tuna, salmon).
• Engage in 30–35 minutes of sun exposure between 11:00 am and 2:00 pm.
• Maintain 7–8 hours of sleep per night.
• Avoid stress, smoking, and alcohol consumption, while maintaining a healthy body weight.
• Perform regular physical activity as part of daily routine.

Therapeutic exercise protocol (TE): ^27– 29^

Both groups will receive therapeutic exercises. Exercises will focus on both back pain and disability minimization of the participants. Each session will last for 25-30 minutes, 4 days per weeks and for 8 weeks. The progression of therapeutic exercise will be as per the registered protocol. Exercises are delivered under a physiotherapist supervision and include:

• Postural advice (TH.1): Maintain erect posture in sitting and standing; avoid prolonged sitting/standing and forward bending.
• McKenzie’s directional preference exercises (TH.2): Sustained positioning and repeated movements, most often extension-based, performed in sets of 10 repetitions.
• Stretching exercises (TH.3): Targeting erector spinae, hamstrings, and triceps surae muscles.
• Lumbar stabilization exercises (TH.4): Core strengthening to enhance spinal stability.
• Weight-bearing aerobic exercises (TH.5): Jogging, stair climbing, and 30 minutes of brisk walking.
• Heating modalities (TH.6): Infra-red radiation applied as adjunct therapy.

Vitamin D supplementation (D):

Participants in the TED group will receive 40,000 IU vitamin D3 capsules once weekly for 8 weeks, prescribed by a registered physician and manufactured by a licensed pharmaceutical company in Bangladesh. ^23^

We expect there will be no major adverse effect for therapeutic exercise and booklet group and that there will be no request of dosage change or worsening of patients’ condition. If any of these occur we will discuss with the patient. If the patient is not willing not to continue, we will stop the intervention, and keep the data for intention to treat analysis. The vitamin D supplement group may experience some adverse effect; we will manage as per the standard measures described in the “safety measures” section. The adherence to these interventions will be monitored through checklist (Extended data 1 ^37^). We will also monitor adverse effect using a checklist (Extended data 2 ^37^).

**Outcome measurement**

***Primary outcome***

*Pain*

The BPI (Brief Pain Inventory), comprising fifteen items, evaluates the degree of pain and how it affects everyday living. It measures pain interference in relationships, emotions, quality of life, and physical activities, including sleep, general activity, and walking; it also contains pain diagrams and questions regarding drugs and analgesics. Higher ratings indicate more acute pain and more interference. ^30^ With an interclass correlation coefficient (ICC) of 0.84–0.90 and kappa values over 0.70, the BPI exhibits great internal consistency (Cronbach's alpha = 0.91) and dependable test–retest results. ^10^

*Vitamin-D3 level*

Serum 25(OH) D will be used to measure the level of Vitamin D3. Tests will be advised by an expert physician, and researchers will collect information from laboratory test reports. Patients will be categorized based on vitamin D levels, such as deficient (less than 20 ng/mL); insufficient (21 to 29 ng/mL); and sufficient (more than 30 to 100 ng/mL). ^10, 31^

***Secondary outcome***

*Disability*

The RMDQ (Roland–Morris Questionnaire) is a 24-item patient-reported instrument that is intended to evaluate pain-related impairment resulting from LBP. Every item has a value of 0 if left blank or 1 if approved, therefore producing a total score between 0 and 24, with higher scores denoting greater impairment. ^32^ Whereas absolute reliability (Standard Error of Measurement, SEM) is estimated between 1.7 and 2.0 points, test-retest reliability usually falls within an intra-class correlation range of 0.79-0.88. ^33^

## Participant timeline

### Sample size

The sample size for this trial was determined based on methodological standards used in low back pain research. Previous studies have indicated that randomized controlled trials in chronic low back pain require a minimum of 152 participants to detect clinically meaningful superiority differences, with a significance level (α) of 0.05, statistical power of 80%, and a 95% confidence interval. Considering potential attrition, we plan to recruit additional participants to ensure adequate statistical power and robustness of findings. ^34, 35^

### Randomization

Researchers plan for hospital-based randomization in both study centers from 1 ^st^ December 2022 to 30 ^th^ May 2023 by sequential random sampling and eligibility screening. Participants were randomly assigned to either the therapeutic exercise plus education booklet (TEB) group or the therapeutic exercise plus vitamin D3 supplementation (TED) group using a computer-generated randomization sequence. Randomization was stratified by study center to ensure balanced distribution across sites. Allocation concealment was maintained through the use of sequentially numbered, opaque, sealed envelopes prepared by an independent researcher not involved in recruitment or assessment.

Blinding was implemented at multiple levels to minimize bias. Outcome assessors were blinded to group allocation throughout the study, ensuring objective evaluation of pain, disability, and serum vitamin D levels. Data analysts were also blinded to group identity during statistical analysis. Due to the nature of the interventions, participants and treating physiotherapists could not be blinded; however, strict separation between intervention delivery and outcome assessment was maintained to preserve methodological rigor.

### Recruitment and study procedure

**Figure 1.**
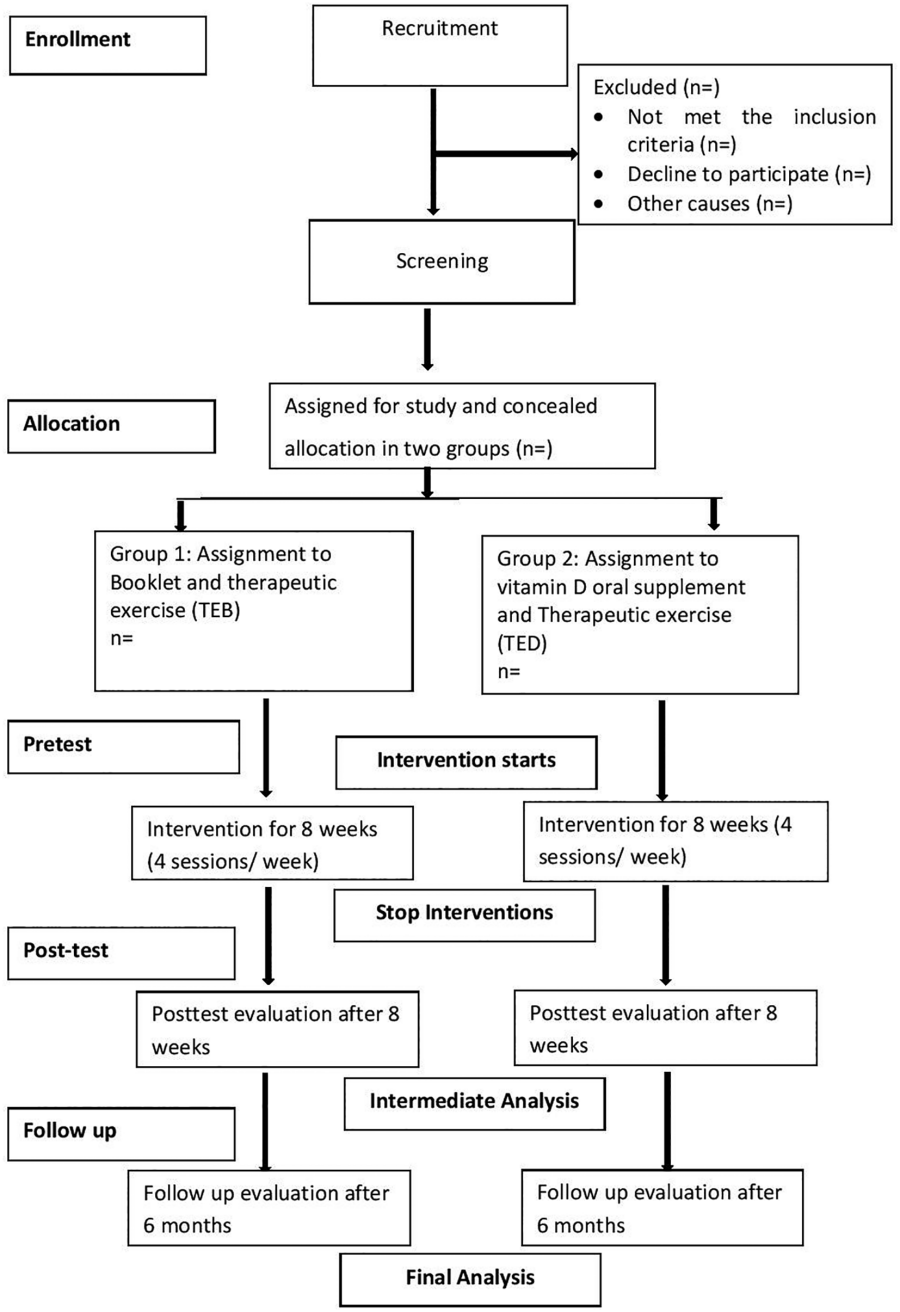
CONSORT diagram.

We will follow the Consolidated Standards of Reporting Trials (CONSORT) to maintain the standards of the study procedure ( Figure 1). For the initial recruitment the LBP patients attending outdoor clinics of both centers from 1st December 2022 to 30th May 2022 will be primarily screened by the outdoor team and provided participant information sheet (PIS) of the study. The final screening will be performed by two licensed physician specializing in physical medicine and rehabilitation. These physicians held postgraduate qualifications (MD/MS in Rehabilitation Medicine or Orthopedics) and had a minimum of 5–10 years of clinical experience in musculoskeletal disorders and pain management. The patient will meet the blinded assessor who will then take pretest data in a separate room, and collect a blood sample, before returning the patient to the outdoor pool. From outdoor pool, the patient will have a concealed envelop with another random ID number matched by the initial ID number given and meet the intervention provider (physiotherapist and physician or physiotherapist alone). Patient will receive the intervention provided in the written guideline enclosed in the concealed envelope. After 8 weeks of treatment completion, the patient will be further screened by blinded assessor, and another blood sample will be collected before discharge. After six months, the patient will be invited to the treatment center or visited in their house or workplace for follow up evaluation and blood sample collection. Therapeutic exercise will be provided by a graduate physiotherapist, and medication will be provided by a registered medical practitioner. Patient will pay for the physiotherapy treatment sessions but will not pay for any additional blood tests, medication or booklet.

### Monitoring

Patients will be monitored during the intervention session, and the medication chart and home exercise checklist (Extended data 1 ^37^) will be maintained for recording the interventions. Patient data will be reviewed by a team from a different organization out of the study setting. The completed forms and questionnaire, along with blood report, will be evaluated by the monitoring team. Any kind of change or modifications to the methodology and intervention protocol will be communicated to the Ethics Committees. The research team will have access to the data and interim results and be in charge of making the final decision to change or end the study, hence carrying out interim analysis.

### Safety measures to avoid harmful effects

Although it is expected that vitamin D3 supplementation and therapeutic exercise will not produce harmful effects on patients, patients should be instructed to inform the physician and physiotherapist if they feel any kind of discomfort (including-gastrointestinal, skin, musculoskeletal problem etc.) after the intervention. Before starting, the physiotherapist and physician will screen patients for any contraindications to intervention. If any serious harmful effects are found, researchers will report this during the final publication. The adverse effects reporting checklist will be provided during intervention (Extended data 2 ^37^).

### Data analysis

Data will be analyzed based on its nature. Calculation and data auditing will be done using Microsoft Excel 2016. Data will be analyzed by SPSS version 23, and R-4.2.1 for Windows. Eligibility for parametric analysis will be checked using bell’s curve, skewness, kurtosis, Kolmogorov–Smirnov test and Shapiro–Wilk test. Continuous variables will be represented by using an arithmetic mean and standard deviation. Categorical data will be represented by percentage (%) and frequency. Baseline characteristics between groups will be compared using chi-square tests for categorical variables and independent t-tests or Mann–Whitney U tests for continuous variables, depending on data distribution. Normality will be assessed using the Shapiro–Wilk test. For within-group comparisons across time points (baseline, 2 months, and 6 months), repeated measures ANOVA will be applied for parametric data, with Greenhouse–Geisser correction if sphericity is violated; the Friedman test will be used for non-parametric data. Between-group differences over time will be analyzed using mixed-model repeated measures ANOVA, which accounts for group × time interactions, or generalized estimating equations (GEE) if assumptions are not met. Post-hoc pairwise comparisons will be adjusted using Bonferroni correction. Effect sizes (Cohen’s d or partial eta squared) will be reported to indicate the magnitude of differences. All analyses will be conducted at a two-tailed alpha level of 0.05, and intention-to-treat principles will be applied to handle missing data.

### Ethical issues and informed consent

According to ethical guidelines, the researchers will abide by the Helsinki declaration. The participants' participation will be entirely voluntary, and they will have the right to withdraw from the trial at any time during the trial. The participants will be assured that participation in or withdrawal from the study will not cause any change to their regular treatment program. Participants will sign the informed consent (Extended data 3 ^37^). The Institute of Physiotherapy Rehabilitation and Research of the Bangladesh Physiotherapy Association (BPA) has provided ethical permission (BPA-IPRR/IRB/06/16/2060) on 16 ^th^ June 2022 to proceed with the study (Extended data 4 ^37^). The trial has been registered with Clinical Trials Registry India ( CTRI/2022/11/047074) (Extended data 5 ^37^). In case of any changes to the protocol, research team will notify to Institutional review board, the trial registry platform and in the later publications. The personal information of the participants will be confidential and stored unanimously in a dataset at the Department of nutrition and food technology at Jashore university of Science & Technology.

### Study status

This study has concluded the assignment of health clinics, training of intervention provider, ethical approval and applied for trial registration. We anticipate beginning this trial on 1 ^st^ December 2022.

## Discussion

There is an increasing concern of LBP and vitamin D deficiency for chronic pain suffers that is leading the working people towards disability and inefficiency to work. ^5^^,^^9^ A non-randomized quasi experimental study ^10^ found therapeutic exercise and vitamin D oral supplementation is effective to reduce pain, replenish vitamin D3 level with short term results. Our study will meet the necessity of randomized systematic evaluation of therapeutic exercises and vitamin D supplement compensation in two different approaches, either by sun exposure, nutrition and healthy lifestyle or by taking oral supplements. We will evaluate outcome in both short term (2 months) and long-term effect after 6 months of stopping the intervention. The experimental group is the therapeutic exercise and booklet group because we assume a positive lifestyle and exercise can replace the role of oral medication supplement, as these were derived as a predictor in observational studies. ^9^

The methodological standard of the proposed trial adheres to the Enhancing the QUAlity and Transparency Of health Research (EQUATOR) guidelines to ensure the rigor of the study. As this is a two tailed hypothesis, we assume any treatment can be superior or both may have similar effect. The similar effect is also a positive finding, because oral vitamin D supplement have some adverse effects if taken for longer durations. ^12^ Moreover, if the study would have four arms including two interventions, a group with only vitamin D supplement and another with therapeutic exercise and a placebo vitamin D supplement, that could ensure true effects. However, researchers had to limit the study considering funding, scope of practice and complicated management issues. As outcome indicators pain and vitamin D3 levels will be used as primary outcomes and disability as secondary outcome, because previous research suggests disability as a consequence. ^36^ BPI measures not only pain severity, but also pain affective interference and pain physical interference, ^10^^,^^30^ that is consistent to the effect of intervention.

The findings of this study may provide important insights into the rehabilitative role of therapeutic exercise in chronic low back pain (CLBP). Beyond its established benefits for musculoskeletal function, exercise may influence the production, absorption, deposition, and overall metabolic function of serum vitamin D, thereby enhancing pain modulation and functional recovery. Evidence suggests that exercise improves vitamin D metabolism and reduces musculoskeletal pain through mechanisms such as modifying sensory neuron excitability and regulating both anti-inflammatory and pro-inflammatory cytokines, ultimately contributing to remission of disability. ^7^ If lifestyle education delivered through the booklet demonstrates positive behavioral changes, this could open new avenues for rehabilitation by promoting self-regulation of vitamin D3 levels and supporting natural remission of CLBP. Moreover, the combined approach of therapeutic exercise and supplementation may offer strategies to reduce recurrence or delay the episodic pattern of pain, adding to the repertoire of non-pharmacological options for CLBP therapy. Overall, these findings highlight the potential to broaden rehabilitative strategies by combining exercise-mediated regulation of vitamin D metabolism with conventional physiotherapy, thereby introducing innovative, evidence-based approaches for the management of chronic low back pain.

## Author contributions

MSI, KMAH, MAZ contributed to Conceptualizing, Planning, Funding Acquisition, Investigation, Administration, Writing (review & editing), and approval. MSH, RP, IKJ, MFK contributed to Investigation, Conceptualizing, Supervision, and review. VR, NAU contributed to Conceptualizing, Writing (review &editing), and approval.

## Data accessibility

### Underlying data

No underlying data are associated with this article.

### Extended data

Mendeley Data: Therapeutic Exercise & Vitamin D for CLBP. https://doi.org/10.17632/d4hf2hjjxr.2. ^37^

This project contains the following extended data:

- Extended data 1: the medication chart and home exercise checklist
- Extended data 2: the adverse effects reporting checklist
- Extended data 3: Informed consent
- Extended data 4: Institutional Review Board (IRB) permission
- Extended data 5: Clinical Trial Registry
- Extended data 6: SPIRIT Checklist

Data are available under the terms of the Creative Commons Attribution 4.0 International license (CC-BY 4.0).

### Reporting guidelines

For generating the protocol, we followed the Interventional Trials 2013 (SPIRIT) guidelines (Table 1), and for the RCT we will follow Consolidated statements for reporting randomized trials (CONSORT) guideline (Figure 1).

Mendeley Data: SPIRIT checklist for ‘Comparison of the effects of therapeutic exercise with either an educational booklet or vitamin-D3 supplement in the management of chronic low back pain: study protocol for an assessor blinded multicenter randomized clinical trial’. https://doi.org/10.17632/d4hf2hjjxr.2. ^37^

Data are available under the terms of the Creative Commons Attribution 4.0 International license (CC-BY 4.0).

## References

[1] Froud R Patterson S Eldridge S : A systematic review and meta-synthesis of the impact of low back pain on people’s lives. *BMC Musculoskelet. Disord.* 2014;15(1):1–4. 24559519 10.1186/1471-2474-15-50 PMC3932512

[2] Hoy D Bain C Williams G : A systematic review of the global prevalence of low back pain. *Arthritis Rheum.* 2012;64(6):2028–2037. 10.1002/art.34347 22231424

[3] Pan CC Simon P Espinoza Orías AA : Lumbar facet joint subchondral bone density in low back pain and asymptomatic subjects. *Skelet. Radiol.* 2020;49(4):571–576. 10.1007/s00256-019-03314-w 31673719 PMC7024659

[4] Briggs AM Straker LM Burnett AF : Chronic low back pain is associated with reduced vertebral bone mineral measures in community-dwelling adults. *BMC Musculoskelet. Disord.* 2012;13(1):1–10. 22458361 10.1186/1471-2474-13-49 PMC3359205

[5] Correia MITD Hegazi RA Higashiguchi T : Evidence-based recommendations for addressing malnutrition in health care: an updated strategy from the feedM. E. Global Study Group. *J. Am. Med. Dir. Assoc.* 2014;15(8):544–550. 10.1016/j.jamda.2014.05.011 24997720

[6] Wu Z Malihi Z Stewart AW : The association between vitamin D concentration and pain: a systematic review and meta-analysis. *Public Health Nutr.* 2018;21(11):2022–2037. 10.1017/S1368980018000551 29559013 PMC10260782

[7] Zadro JR Shirley D Ferreira M : Is Vitamin D Supplementation Effective for Low Back Pain? A Systematic Review and Meta-Analysis. *Pain Physician.* 2018;21(2):121–145. 29565945

[8] Hao-Wei X Shu-Bao Z Yu-Yang Y : Relationship between vitamin D and nonspecific low back pain may be mediated by inflammatory markers. *Pain Physician.* 2021;24(7):1015. 34704712

[9] Ali M Uddin Z : Factors associated with vitamin D deficiency among patients with musculoskeletal disorders seeking physiotherapy intervention: a hospital-based observational study. *BMC Musculoskelet. Disord.* 2022;23(1):1–9. 36042435 10.1186/s12891-022-05774-z PMC9426039

[10] Ali M Uddin Z Hossain A : Combined Effect of Vitamin D Supplementation and Physiotherapy on Reducing Pain Among Adult Patients With Musculoskeletal Disorders: A Quasi-Experimental Clinical Trial. *Front. Nutr.* 2021;8:717473. 10.3389/fnut.2021.717473 34676231 PMC8523800

[11] Gordon R Bloxham S : A systematic review of the effects of exercise and physical activity on non-specific chronic low back pain. *In Healthcare.* 2016;4(2):22. 10.3390/healthcare4020022 27417610 PMC4934575

[12] Pardo GB Girbés EL Roussel NA : Pain neurophysiology education and therapeutic exercise for patients with chronic low back pain: a single-blind randomized controlled trial. *Arch. Phys. Med. Rehabil.* 2018;99(2):338–347. 10.1016/j.apmr.2017.10.016 29138049

[13] Holick MF : Sunlight and vitamin D for bone health and prevention of autoimmune diseases, cancers, and cardiovascular disease. *Am. J. Clin. Nutr.* 2004;80(6):1678–1688. 10.1093/ajcn/80.6.1678S 15585788

[14] Simula AS Jenkins HJ Holopainen R : Transcultural adaption and preliminary evaluation of “understanding low back pain” patient education booklet. *BMC Health Serv. Res.* 2019;19(1):1–1. 31888605 10.1186/s12913-019-4854-y PMC6936060

[15] Augustine LF Madhavan KN Kulkarni B : Optimal duration of sun exposure for adequate cutaneous synthesis of vitamin D in Indian cities: an estimate using satellite-based ultraviolet index data. *Biomed. J. Sci. Tech. Res.* 2018;6:5073–5077.

[16] Lakkireddy M Karra ML Patnala C : Efficiency of vitamin D supplementation in patients with mechanical low back ache. *J. Clin. Orthop. Trauma.* 2019;10(6):1101–1110. 10.1016/j.jcot.2019.06.018 31708636 PMC6834986

[17] Sha S Chen LJ Brenner H : Serum 25-Hydroxyvitamin D Status and Vitamin D Supplements Use Are Not Associated with Low Back Pain in the Large UK Biobank Cohort. *Nutrients.* 2024;16(6):806. 38542718 10.3390/nu16060806 PMC10974643

[18] Zadro J Shirley D Ferreira M : Mapping the association between vitamin D and low back pain: a systematic review and meta-analysis of observational studies. *Pain Physician.* 2017;20(7):611–640. 29149142

[19] Zaman S Hawlader MD Biswas A : High prevalence of vitamin D deficiency among Bangladeshi children: an emerging public health problem. *Health.* 2017;09(12):1680–1688. 10.4236/health.2017.912123

[20] Ghai B Bansal D Kanukula R : Vitamin D supplementation in patients with chronic low back pain: an open label, single arm clinical trial. *Pain Physician.* 2017;20(1):E99–E105. 10.36076/ppj.2017.1.E99 28072801

[21] Akhtar RR Ahmed R Ashraf S : Effect of Vitamin-D supplementation in adults presenting with chronic lower back pain. *J. Rawalpindi Med. Coll.* 2020;24(2):161–165.

[22] Lee TJ Tsai RY Ho CC : Updated Meta-analysis Reveals Limited Efficacy of Vitamin D Supplementation in Chronic Low Back Pain. *In vivo.* 2024;38(6):2955–2967. 39477425 10.21873/invivo.13778 PMC11535934

[23] Islam MS Zahid MA Hossain MS : Effect of Therapeutic Exercise, Educational booklet and Vitamin D3 Supplement for the Management of Chronic Mechanical Low Back Pain Muhammad Shahidul Islam, Dr. Md. Ashrafuzzaman Zahid, Professor Dr. Md. Sohrab Hossain, protocols.io. 2022. 10.17504/protocols.io.rm7vzyqz5lx1/v1 PMC13044536 41937888

[24] Gao Q Kou T Zhuang B : The association between vitamin D deficiency and sleep disorders: a systematic review and meta-analysis. *Nutrients.* 2018;10(10):1395. 10.3390/nu10101395 30275418 PMC6213953

[25] Dean E Söderlund A : What is the role of lifestyle behaviour change associated with non-communicable disease risk in managing musculoskeletal health conditions with special reference to chronic pain? *BMC Musculoskelet. Disord.* 2015;16(1):1–7. 25888381 10.1186/s12891-015-0545-y PMC4397667

[26] Kim D Cho M Park Y : Effect of an exercise program for posture correction on musculoskeletal pain. *J. Phys. Ther. Sci.* 2015;27(6):1791–1794. 10.1589/jpts.27.1791 26180322 PMC4499985

[27] Dunsford A Kumar S Clarke S : Integrating evidence into practice: use of McKenzie-based treatment for mechanical low back pain. *J. Multidiscip. Healthc.* 2011;4:393. 22135496 10.2147/JMDH.S24733 PMC3215349

[28] França FR Burke TN Caffaro RR : Effects of muscular stretching and segmental stabilization on functional disability and pain in patients with chronic low back pain: a randomized, controlled trial. *J. Manip. Physiol. Ther.* 2012;35(4):279–285. 10.1016/j.jmpt.2012.04.012 22632587

[29] Yoon JS Lee JH Kim JS : The effect of swiss ball stabilization exercise on pain and bone mineral density of patients with chronic low back pain. *J. Phys. Ther. Sci.* 2013;25(8):953–956. 10.1589/jpts.25.953 24259892 PMC3820231

[30] Farrar JT Young JP Jr LaMoreaux L : Clinical importance of changes in chronic pain intensity measured on an 11-point numerical pain rating scale. *Pain.* 2001;94(2):149–158. 10.1016/S0304-3959(01)00349-9 11690728

[31] Harinarayan CV Holick MF Prasad UV : Vitamin D status and sun exposure in India. *Dermato-endocrinology.* 2013;5(1):130–141. 10.4161/derm.23873 24494046 PMC3897581

[32] Jordan K Dunn KM Lewis M : A minimal clinically important difference was derived for the Roland-Morris Disability Questionnaire for low back pain. *J. Clin. Epidemiol.* 2006;59(1):45–52. 10.1016/j.jclinepi.2005.03.018 16360560

[33] Islam SM Emran M Baral AB : Roland Morris disability questionnaire in Bengali for evaluation of patients with low back pain. *KYAMC J.* 2020;11(1):21–25. 10.3329/kyamcj.v11i1.47146

[34] Froud R Rajendran D Patel S : The power of low back pain trials: a systematic review of power, sample size, and reporting of sample size calculations over time, in trials published between 1980 and 2012. *Spine.* 2017;42(11):E680–E686. 10.1097/BRS.0000000000001953 27792111

[35] Ostelo RW Deyo RA Stratford P : Interpreting change scores for pain and functional status in low back pain: towards international consensus regarding minimal important change. *Spine.* 2008;33(1):90–94. 10.1097/BRS.0b013e31815e3a10 18165753

[36] Gallagher KM Campbell T Callaghan JP : The influence of a seated break on prolonged standing induced low back pain development. *Ergonomics.* 2014;57(4):555–562. 10.1080/00140139.2014.893027 24734970

[37] Hossain KMA Zahid MA Islam MS : Therapeutic Exercise & Vitamin D for CLBP. *Mendeley Data.* 2022;V2. 10.17632/d4hf2hjjxr.2

