# Supplementary material for: Comparison of the effects of therapeutic exercise with either an educational booklet or vitamin-D3 supplement in the management of chronic low back pain: study protocol for an assessor blinded multicenter randomized clinical trial
Source: F1000Res. 2026 Mar 3;11:1352. Originally published 2022 Nov 21. [Version 2] doi: 10.12688/f1000research.127948.2 (PMC13044536; doi:10.12688/f1000research.127948.2)
Supplement: Peer review history

# Reviewer report: Reviewer response for version 2

**DOI:** 10.5256/f1000research.196043.r466508

**Published:** 2026-04-01

**Authors:** Mohammad Sidiq, Akansha Suman

**Affiliations:** 1 Tishk International University, Erbil, Iraq; 2 Department of Physiotherapy, School of Paramedical & Allied Health Sciences, Shri Guru Ram Rai University, Dehradun, Uttarakhand, India

I would like to appreciate authors for this proposed trial but it needs some amendments as under:

 This protocol outlines a randomized trial to compare the effectiveness of two intervention strategies to treat chronic low back pain (CLBP) in individuals with low vitamin D levels. The purposes of the trial are to compare the impact of therapeutic exercises in the context of either pain neuroscience education or vitamin D supplementation on pain, function, and other relevant outcomes of interest.

 The study protocol employs a randomized, assessor-blinded, multicenter, parallel-group design, which effectively addresses the research question. The research is of interest because of the high prevalence of CLBP and growing interest in both mind-related (education) and body-related (vitamin D) issues.

 The study addresses a significant gap, but there are areas where details of the study methodology and reporting need to be clarified for proper replication, reproduction, and rigor.

 Major Comments (Must Be Addressed)

 1. Explanation of Research Gap

 The justification for the study is explained, but not why the research gap exists. Insufficient detail is provided in the manuscript on:

 •    That evidence is insufficient

 •    The contribution this new study makes compared to previous trials

 What's more, the objectives are not well prioritized:

 •    The primary outcome must be identified

 •    Secondary outcomes must be identified

 Recommendation:

 Make sure to clearly justify and identify novelty in the introduction. Separately state what is primary vs secondary

 2. Sample Size

 The protocol is lacking a sound sample size calculation, which is an important design consideration.

 Recommendation:

 Include:

 •    Estimated effect size (citation)

 •    Power (typically 80–90%)

 •    Alpha level

 •    Estimated dropout rate

 •    Estimated sample size

 3. Methods of Randomization and Allocation Concealment

 Randomization is stated, but specific details are lacking, such as:

 •    Randomization sequence generator (e.g., computer)

 •    Mechanism used to conceal allocation (e.g., opaque sealed envelopes)

 Recommendation:

 Describe allocation of groups in detail and according to CONSORT to reduce bias in selection.

 4. Interventions (To enable repetitive studies)

 General but little detail about the interventions:

 •    Exercise type, intensity, progression and supervision

 •    Pain neuroscience education content and format

 •    Dose, frequency, duration and reason for vitamin D supplement

 •    Adherence and compliance monitoring

 Recommendation:

 Include more detail on intervention using methods such as TIDieR

 5. Statistical Analysis Plan

 The plan lacks sufficient specificity regarding the statistical analysis.

 •    Approaches to missing data (e.g., intention-to-treat)

 •    Adjustment for baseline covariates

 •    Adjustment for baseline covariates

 •    Subgroup or "post-hoc" analyses

 Recommendation:

 Offer a full statistical analysis plan for greater transparency.

 6. Outcomes and timepoints

 Even though outcomes are discussed, the protocol does not include:

 •    clear specification of a primary outcome measure

 •    Tabulated display of timepoints for assessments

 •    Rationale for choice of outcomes

 Recommendation:

 Provide a table that includes:

 •    Primary and secondary outcomes

 •    Measurement tools

 •    Time (baseline, follow-up)

 You can also include MCID.

 Minor Comments (Should Be Addressed)

 1. Blinding and Bias

 •    The assessors were blinded

 •    But participants cannot be blinded, and this introduces bias

 See discussion of performance and expectation bias.

 2. Confounding Variables

 Potential confounders are not controlled for:

 •    Sunlight exposure

 •    Dietary vitamin D intake

 •    Levels of physical activity

 Add measures to either control for these variables or plan for recording them.

 3. Ethical Considerations

 Consent is sought, but:

 •    Ensure safety monitoring is detailed for vitamin D supplements

 Include procedures for safety monitoring and adverse events

 4. Trial Registration

 • Take care to ensure the trial number is clearly identified.

 5. Language and Structure

 •    Some grammatical improvements needed

 •    Some improvements to be made for clarity

 Overall Recommendation

 Major Revision

 The protocol poses a relevant and timely clinical question, suitable for a randomized controlled evaluation. But important study details are missing, particularly in terms of sample size, study intervention, outcome measures, and statistical considerations. This is important to ensure the study is rigorously conducted, replicable, and meets international reporting guidelines.

Is the study design appropriate for the research question?

Yes

Is the rationale for, and objectives of, the study clearly described?

Partly

Are sufficient details of the methods provided to allow replication by others?

Partly

Are the datasets clearly presented in a useable and accessible format?

Not applicable

Reviewer Expertise:

Musculoskeletal, Pain Sciences and Public Health

We confirm that we have read this submission and believe that we have an appropriate level of expertise to confirm that it is of an acceptable scientific standard, however we have significant reservations, as outlined above.

# Reviewer report: Reviewer response for version 2

**DOI:** 10.5256/f1000research.196043.r464590

**Published:** 2026-03-25

**Authors:** Spyridon Sotiropoulos

**Affiliations:** 1 University of West Attica, Athens, Greece

This manuscript presents a study protocol for a two-arm, assessor-blinded randomized clinical trial investigating the additional effect of vitamin D3 supplementation compared with an educational booklet when combined with therapeutic exercise in individuals with chronic low back pain and vitamin D deficiency. The topic is clinically relevant, given the high prevalence of both low back pain and hypovitaminosis D, and the growing interest in multimodal management strategies.

 The rationale and objectives are clearly described. The authors provide a coherent background linking vitamin D deficiency with musculoskeletal pain, and they articulate a clear hypothesis alongside well-defined primary and secondary objectives. Although the background appropriately acknowledges that existing evidence is mixed, some statements may still lean toward overstating the potential benefits of vitamin D supplementation. Nonetheless, the justification for conducting the trial is reasonable and clearly presented.

 The overall study design—a randomized, assessor-blinded trial—is appropriate for the research question and reflects a pragmatic approach consistent with physiotherapy practice. However, a key methodological concern remains insufficiently addressed. The allocation of different intervention arms to separate centers introduces a risk of confounding between treatment and study site. Differences in patient populations, clinician behavior, or contextual factors across centers may influence outcomes independently of the intervention. While the authors note stratification by study center, this approach does not fully resolve the issue if intervention and center are structurally linked. The manuscript would be strengthened by explicitly describing how site effects will be accounted for in the statistical analysis (e.g., inclusion as a fixed or random effect) or by providing a stronger justification for this design choice.

 The methods are described in substantial detail and are generally sufficient to support replication. Eligibility criteria, intervention protocols, outcome measures, and timelines are clearly specified. The inclusion of assessor and data analyst blinding is a strength. Adherence to interventions is addressed through the use of medication charts and exercise checklists, and adverse events are monitored systematically. The statistical analysis plan is also well developed, including intention-to-treat principles, appropriate modeling approaches, and reporting of effect sizes. However, further clarification on the handling of site-related effects within the analysis would improve methodological transparency.

 As expected for a study protocol, no dataset is presented. The authors provide comprehensive supporting materials, including checklists, consent forms, and trial documentation, which enhances transparency and reproducibility. A brief statement outlining how and when the final dataset will be shared would further strengthen alignment with open science practices.

 In summary, this is a well-structured and clinically relevant protocol with generally strong methodological detail. The primary issue requiring attention is the potential for center-related confounding due to the allocation of interventions across sites. Addressing this concern would strengthen the internal validity of the study. Secondary improvements relate to clarifying data-sharing plans. These issues are addressable and do not fundamentally undermine the overall study design.

Is the study design appropriate for the research question?

Yes

Is the rationale for, and objectives of, the study clearly described?

Yes

Are sufficient details of the methods provided to allow replication by others?

Yes

Are the datasets clearly presented in a useable and accessible format?

Yes

Reviewer Expertise:

Musculoskeletal physiotherapy, chronic low back pain, exercise therapy, non-pharmacological interventions, clinical trial methodology and rehabilitation research.

I confirm that I have read this submission and believe that I have an appropriate level of expertise to confirm that it is of an acceptable scientific standard.

# Reviewer report: Reviewer response for version 1

**DOI:** 10.5256/f1000research.140494.r181246

**Published:** 2023-06-29

**Authors:** Gopal Nambi

**Affiliations:** 1 Department of Health and Rehabilitation Sciences, College of Applied Medical Sciences, Prince Sattam Bin Abdulaziz University, Alkharj, Saudi Arabia

Dear Authors

 Thanks a lot for the opportunity you have offered me to revise the fascinating manuscript, "Comparison of the effects of therapeutic exercise...randomized clinical trial". I thank the authors for their effort in producing this exciting manuscript.

 From the editing point of view, I recommend the authors to fully respect the editing requirements imposed by this scientific journal and clearly indicated in the template. More specifically, I mean: the number of words in the abstract and manuscript, the number of keywords and the way to indicate the bibliographic sources.

 As a significant strength: This proposal is a novelty in the field and adds information to the existing evidence in the literature produced in the field.

 As a major weakness: The manuscript lacks details and clarity concerning methodological steps that would help improve the understanding of the manuscript.

 Keywords: use MeSH keywords

 Abstract:

1. The background of the study talks only about CLBP.
2. The objective of the study is not clear.
3. Mention the study duration and study setting.
4. Mention the character of the study participants.
5. Mention the treatment procedure in short.
6. Mention the outcome measures in short and its duration of measurement.
7. Mention the statistical tests used for the study.

Manuscript

1. Mention the acronym of abbreviation when it is used for the first time.
2. Please recheck the reference number 5.
3. Mention the relation between CLBP and vitamin D deficiency in detail.
4. Mention in detail about Vit D supplement, its role, merits and demerits in CLBP.
5. How come this study is differing from the reference study 10?
6. Mention the gaps monitored by the researcher in the previous studies.
7. Mention the study hypothesis.
8. Include the clinical significance of this study over clinicians, patients, and researchers after the study hypothesis.
9. Mention who has diagnosed the participants and their qualification and experience.
10. Mention the detail information about randomization, allocation and blinding.
11. Mention in detail about the exercise protocol and the contents involved in booklet.
12. Mention the outcome measures measured in the study and its reliability and validity.
13. Mention the sample size calculation with reference. The information provided is not sufficient.
14. Mention the statistical tests included in the study. The tests mentioned are not suitable.

Is the study design appropriate for the research question?

Partly

Is the rationale for, and objectives of, the study clearly described?

No

Are sufficient details of the methods provided to allow replication by others?

No

Are the datasets clearly presented in a useable and accessible format?

Partly

Reviewer Expertise:

Musculoskeletal and sports physiotherapy

I confirm that I have read this submission and believe that I have an appropriate level of expertise to state that I do not consider it to be of an acceptable scientific standard, for reasons outlined above.

# Response

**Published:** 2026-01-31

**Authors:** Dr. Kazi Md. Amran Hossain, PT

**Reviewer 2**

**Comment 1:** From the editing point of view, I recommend the authors to fully respect the editing requirements imposed by this scientific journal and clearly indicated in the template. More specifically, I mean: the number of words in the abstract and manuscript, the number of keywords and the way to indicate the bibliographic sources.

**Response:** We thank the reviewer for this valuable observation and have carefully revised the manuscript to ensure full compliance with the journal’s editorial requirements. The abstract has been shortened to meet the word limit, the number of keywords has been adjusted according to the journal’s specifications, and all bibliographic references have been reformatted to strictly follow the prescribed citation style. In addition, we verified that the overall manuscript length now falls within the required limits. We believe these revisions address the reviewer’s concern and ensure that the manuscript fully respects the journal’s formatting and editing standards.

**Comment 2:** As a significant strength: This proposal is a novelty in the field and adds information to the existing evidence in the literature produced in the field.

**Response:** We sincerely appreciate the reviewer’s recognition of the novelty and contribution of our proposal. We agree that the study introduces an innovative perspective in the field and adds valuable information to the existing body of evidence. By addressing current gaps and integrating findings with the broader literature, we believe our work strengthens the scientific understanding and provides meaningful insights that can guide future research and clinical practice.

**Comment 3: **As a major weakness: The manuscript lacks details and clarity concerning methodological steps that would help improve the understanding of the manuscript.

**Response:** We thank the reviewer for this constructive observation and acknowledge the importance of providing clear methodological details to enhance the transparency and reproducibility of our work. In the revised manuscript, we have expanded the description of the methodological steps, including participant selection criteria, intervention procedures, outcome measures, and statistical analyses. These additions aim to improve clarity and allow readers to better understand the study design and implementation. We believe that the revisions now address the reviewer’s concern and strengthen the methodological rigor and comprehensibility of the manuscript.

**Comment 4: **

- Keywords: use MeSH keywords

Abstract:

- The background of the study talks only about CLBP.
- The objective of the study is not clear.
- Mention the study duration and study setting.
- Mention the character of the study participants.
- Mention the treatment procedure in short.
- Mention the outcome measures in short and its duration of measurement.
- Mention the statistical tests used for the study.

**Response: **We thank the reviewer for these constructive suggestions regarding the abstract and keywords. In the revised manuscript, we have replaced the keywords with appropriate MeSH terms to ensure consistency with indexing standards. The abstract has been thoroughly revised to provide a clearer and more comprehensive overview of the study. Specifically, the background now contextualizes the research beyond chronic low back pain (CLBP), the study objective has been explicitly stated, and details regarding the study duration and setting have been included. We have also described the characteristics of the study participants, summarized the treatment procedure, and briefly outlined the outcome measures along with the timing of their assessment. Finally, the statistical tests employed in the analysis have been mentioned to enhance transparency. We believe these revisions improve the clarity and completeness of the abstract and align it more closely with the journal’s requirements.

**Comment 5: **How come this study is differing from the reference study 10?

**Response: **We appreciate the reviewer’s observation regarding the differences between our study and Reference 10. The study by Ali et al. (2021) investigated the combined effect of vitamin D supplementation and physiotherapy in patients with musculoskeletal disorders using a quasi-experimental design. In contrast, our study specifically focuses on chronic low back pain and employs a randomized, assessor-blinded, multicenter clinical trial design to compare the effects of therapeutic exercise when combined either with an educational booklet or with vitamin D3 supplementation. Thus, while both studies examine the role of vitamin D in musculoskeletal pain, our trial differs in its methodological rigor, target population, and intervention comparison, aiming to provide higher-level evidence on the relative effectiveness of exercise combined with different adjuncts in CLBP management.

**Comment 6: **

- Mention the gaps monitored by the researcher in the previous studies.
- Mention the study hypothesis.
- Include the clinical significance of this study over clinicians, patients, and researchers after the study hypothesis.
- Mention who has diagnosed the participants and their qualification and experience.
- Mention the detail information about randomization, allocation and blinding.
- Mention in detail about the exercise protocol and the contents involved in booklet.
- Mention the outcome measures measured in the study and its reliability and validity.
- Mention the sample size calculation with reference. The information provided is not sufficient.
- Mention the statistical tests included in the study. The tests mentioned are not suitable.

**Response:** We thank the reviewer for these constructive suggestions. In the revised manuscript, we addressed the issues throughout the manuscript. Thank you.

# Reviewer report: Reviewer response for version 1

**DOI:** 10.5256/f1000research.140494.r181251

**Published:** 2023-06-28

**Authors:** Giacomo Farì

**Affiliations:** 1 Department of Basic Sciences, Neuroscience and Sense Organs, Aldo Moro University of Bari, Bari, Italy

This protocol is interesting and in line with the aims of this journal. Some concerns have to be addressed.

 I really appreciate that you provided the clinical trial registration and the IRB approval, but it is still not clear if this study will be a prospective or a retrospective one?

 Then, since you compare three interventions, very different from each other, you should better define the inclusion/exclusion criteria: which Low back pain will you consider? Which diseases will be excluded and which ones no?

 Finally, to improve the rehabilitative lapels of this study, you should explain in the discussion how your results could add new options for chronic low back pain therapy and rehabilitation. 

 Best regards and good luck

Is the study design appropriate for the research question?

Partly

Is the rationale for, and objectives of, the study clearly described?

Yes

Are sufficient details of the methods provided to allow replication by others?

Yes

Are the datasets clearly presented in a useable and accessible format?

Yes

Reviewer Expertise:

rehabilitation

I confirm that I have read this submission and believe that I have an appropriate level of expertise to confirm that it is of an acceptable scientific standard, however I have significant reservations, as outlined above.

# Response

**Published:** 2026-01-31

**Authors:** Dr. Kazi Md. Amran Hossain, PT

**Reviewer 1**

 This protocol is interesting and in line with the aims of this journal. Some concerns have to be addressed.

**Comment 1: **I really appreciate that you provided the clinical trial registration and the IRB approval, but it is still not clear if this study will be a prospective or a retrospective one?

**Response:** Thank you for your valuable comment. It is indeed a prospective study. I have completed the baseline and post intervention data collection now we are collecting the follow-up information of the participants. 

**Comment 2:** Then, since you compare three interventions, very different from each other, you should better define the inclusion/exclusion criteria: which Low back pain will you consider? Which diseases will be excluded and which ones no?

**Response:** Thank you for your valuable comment. We have rewritten the exclusion criteria subsection of the manuscript according to your suggestions.

**Comment 3:** Finally, to improve the rehabilitative aspects of this study, you should explain in the discussion how your results could add new options for chronic low back pain therapy and rehabilitation.

**Response:** We appreciate the reviewer’s suggestion to clarify how our findings could contribute to rehabilitation strategies for chronic low back pain. In the revised discussion, we have emphasized the potential rehabilitative implications of our study. Specifically, we highlight that therapeutic exercise may not only reduce musculoskeletal pain and disability but also play a role in regulating vitamin D metabolism, thereby offering a novel mechanism for functional recovery. We further explain that lifestyle education, if effective, could promote self‑regulation of vitamin D3 levels and support natural remission of CLBP. Thank you.
